# Spindle cell epithelioma of the vagina with rare morphology: a case report and literature review

**DOI:** 10.3389/fonc.2025.1551607

**Published:** 2025-08-04

**Authors:** Xian-bin Tang, Yuan-gui Xu, Li Yao, Ling-ling Yuan, Yu-gang Huang

**Affiliations:** ^1^ Department of Pathology, Taihe Hospital, Hubei University of Medicine, Shiyan, China; ^2^ Department of Pathology, Zhuxi People’s Hospital, Shiyan, China

**Keywords:** spindle cell epithelioma of the vagina, immunohistochemistry, diagnostic pathology, gynecological pathology, case report

## Abstract

Spindle cell epithelioma of the vagina (SCEV) is a rare female genital tract neoplasm with a complex morphology and immunophenotype easily resulting in misdiagnosis. The tumor was primarily composed of spindle and epithelioid cells. In this case, there was no obvious epithelial component in the tumor parenchyma, and only epithelioid cells with rounded nuclei were observed, which were tightly mixed with the spindle cells. Here, we describe a unique case of vaginal spindle cell epithelioma in a 51-year-old woman. In this tumor, mucoid degeneration and the formation of vacuoles or microcysts with varying sizes without epithelial lining were observed, and only a few glandular structures were observed in the periphery of the tumor. Immunohistochemical staining showed diffuse positivity for AE1/AE3, CK7, and CD10 in both spindle and epithelioid cells, and ER, PR, P63, and P40 were diffusely expressed in the spindle cells, while expressed to varying extents in epithelioidal cells. CD34 was expressed focally in spindle cells. No expression of Desmin, SMA, CD31, Caldesmon, TLE-1, WT-1, and SS18 was in the tumor, which could be differentiated from synovial sarcoma. Tumor cells were negative for S100 and positive for CD10 and BCL2 in their mesenchyme. There are conflicting reports on the tissue origin of SCEV, and the immunohistochemical staining of this case supports the hypothesis that SCEV originates from multipotent stem cells.

## 1 Introduction

Spindle cell epithelioma of the vagina (SCEV), also known as vaginal mixed tumor, were first reported by Brown in 1953 (1). SCEV is most common found in women aged 40 to 50 years (2) and is often detected incidentally; the tumor is mostly located in the lower part of the vagina near the hymenal ring, and can occasionally be found in other parts of the vagina (2), which is one of the prominent features of the tumor. Since it is also a common site for vaginal polyps or cysts, it is often misdiagnosed as a vaginal polyp or cyst.

## 2 Case presentation

### 2.1 Clinical information

The patient was a female, 51 years old. She was admitted to the hospital because of uterine fibroids that had been detected for 2 years. Ultrasound examination suggested that the myometrium of the posterior wall of the lower uterine segments bulged outward, and a hypoechoic mass of 76 mm× 73 mm× 63 mm was observed. Other gynecological examinations revealed a small cyst near the vaginal opening. Two days after admission, the patient underwent laparoscopic total hysterectomy, bilateral adnexectomy, and cystectomy of the left vaginal wall. The cyst in the vaginal opening is adjacent to the remnants of the hymen.

### 2.2 Immunohistochemistry

The immunohistochemical primary antibodies involved in this study, including AE1/AE3, CK7, ER, RP, CD10, BCL2, CD34, S100, SMA, Desmin, WT-1, SS18, TLE-1, P63, P40, CD31, and Ki67 were purchased from DAKO company with ready-to-use, and the secondary antibody was K-8002 from DAKO. The immunostaining was performed with Link-48 (DAKO) platform.

## 3 Results

### 3.1 Macroscopy

A cyst measuring 1cm× 0.4 cm was found in the specimen of vaginal wall.

### 3.2 Microscopic morphology

**Figure 1:**
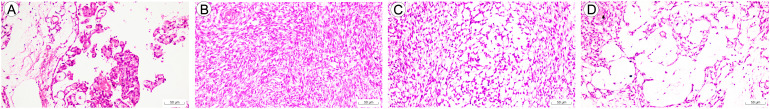
Morphology of spindle cell epitheliomas of the vagina (SCEV). **(A)** The periphery of the tumor shows a cystic cavity with adenoidal and papillary structures, and the papillae are lined with cuboidal epithelium (HE; high power fields, 200×; scale bar, 50 μm). **(B)** Solid areas of spindle-shaped tumor cells arranged in cross-bunches (HE; high power fields, 200×; scale bar, 50 μm). **(C)** Vacuoles and microcapsules are distributed singly or in patches between spindle cells to form a reticular structure (HE; high power fields, 200×; scale bar, 50 μm). **(D)** Zone of sparse cellularity in the internal partitions of the tumor with interstitial edema. (HE; high power fields, 200×; scale bar, 50 μm).

Most areas of the tumor were solid and adjacent to a cystic structure which was filled with adenoidal and papillary structures lined by epithelioid tumor cells (**Figure 1A**). The cells in the solid area were mainly composed of short spindle cells with some of epithelioid cells, and the spindle cells were arranged in a cross-bundle (**Figure 1B**). A large number of small vacuolated, microcystic structures could be seen between the tumor cells, which were aggregated in a single or sheet-like manner to form a reticulum (**Figure 1C**). The distribution of the tumor cells was uneven, and edematous changes were observed in the area short of tumor cells (**Figure 1D**). Fibrous nodules with vitreous degeneration were scatteringly distributed in the tumor. Multifocal mucinous changes were seen within the tumor parenchyma; squamous cell clusters and glands lined with epithelium were not observed. The tumor cells were morphologically mild with slightly eosinophilic cytoplasm. Nuclear heterogeneity and mitosis were not observed.

### 3.3 Immunohistochemistry

**Figure 2:**
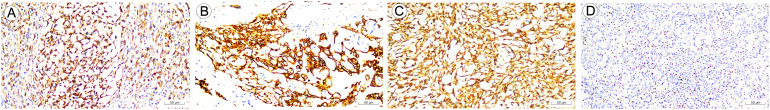
Immunohistochemical staining of biomarkers in spindle cell epitheliomas of the vagina (SCEV). **(A)** Uneven staining intensity of AE1/AE3, with more positive intensity in the reticular area than in the solid area (IHC, EnVision; high power fields, 200×; scale bar, 50 μm). **(B)** CD10 is diffusely and strongly positively expressed in adenoepithelial cells (IHC, EnVision; high power fields, 200×; scale bar, 50 μm). **(C)** CD10 spindle cells showed diffuse strong positive expression (IHC, EnVision; high power fields, 200×; scale bar, 50 μm). **(D)** P40 is diffusely positively expressed in spindle cells (IHC, EnVision; high power fields, 200×; scale bar, 50 μm).

AE1/AE3 demonstrated diffuse cytoplasmic immunoreactivity, stronger in the reticular zone than in the solid zone (**Figure 2A**), with the strongest expression in the intracapsular glands; CK7 expression pattern was similar to that of AE1/AE3; BCL-2 was moderately diffusely positive in cytoplasm in the adenoidal zone and in the spindle cell zone; ER was diffusely strongly positive in nuclei in the spindle cells, while weakly positive and scattered in the epithelioid cell areas, PR was diffusely weakly nuclear- positive in the spindle cells and negative in epithelioid cells; Cytoplasmic positivity for CD10 is diffuse in both spindle and epithelioid cells (**Figures 2B, C**); Nuclear expression of P63 was focally moderately strong positive in spindle cells and negative in epithelioid cells; Nuclear positivity for P40 was diffuse in spindle cells (**Figure 2D**) and scattered in epithelioid cells; Index of K-i67 of the tumor was in approximately 1%-2%; CD34 was focally positive in the tumor. Desmin, SMA, S100, CD31, Caldesmon, TLE-1, WT-1 and SS18 were negative in the tumor.

## 4 Discussion

The most distinctive histological signature of vaginal spindle cell epithelioma is a close mixture of epithelial and mesenchymal components (3), where the epithelial component is the glandular epithelium, squamous epithelium, or both (4). The squamous cell component may be more differentiated and intercellular bridges may be observed (3). However, a glandular component was identified only at the tumor margins in this case, characterized by glandular lumens lined with cuboidal epithelial cells, similar to that reported by Berdugo et al (5). Squamous components were not found. The immunophenotypes of the glandular lining epithelium and the spindle cells in the solid component were mostly similar, supporting that both were part of the tumor. The arrangement of spindle cells within the tumor is pattern less and display an uneven distribution. Areas rich in cells resemble smooth muscle or endometrial mesenchymal tumors. Notably, mucus was identified between cells in more sparsely cellular regions, resulting in vacuoles of varying sizes and adenoid structures. However, only light-stained mucus was seen in the lumen without lining the epithelium, thus a pseudo-glandular lumen, which is a striking morphological feature of this case.

Although the immunohistochemical profile of vaginal spindle cell epithelial tumors is complex and variable, epithelial and mesenchymal cells almost always diffusely express CD10, AE1/AE3, ER, and PR (5–7). CD10 is a sensitive marker for endometrial mesenchymal tumors, but it is also highly sensitive for SCEV (7). Oliva E (6) et al. reported that all 13 cases of SCEV were positive for CD10; thus, the diffuse and strong expression of CD10 is suggestive in the diagnosis of SCEV, but it needs to be distinguished from metastatic or primary endometrial stromal tumors in the vagina (8); Although there are a few early reports in the literature that endometrial mesenchymal tumors express epithelial markers (9, 10), the majority of endometrial mesenchymal sarcomas do not express epithelial markers, so the co-expression of CD10 and CK is more supportive of the diagnosis of SCEV; in the present case, there was no obvious epithelial component in the parenchyma of the tumor, and only epithelioid cells with rounded nucleic closely mixed with spindle cell were observed, resulting difficult differentiation from endometrial mesenchymal tumors or smooth muscle tumors. But, the diffuse expression of P63 and P40 could exclude the above two types of tumors; contrary to the report of Xie Jianjun et al. (11), the intensity and range of the expression of P40 and P63 in spindle cells were significantly higher than those in adenoepithelial cells. It has also been reported that P63 is negative in SCEV (12, 13). Myogenic markers have a high positive rate in other reports (5, 6), but in this case, SMA, Desmin, and Calsesmon were negatively expressed, which did not support smooth muscle or myoepithelium origin.S100 is rarely positively expressed in SCEV, and only one case has been reported so far (6); in this case, S100 expression was negative, which further excluded the possibility of myoepithelium origin. WT1 was strongly and diffusely positive in two cases of SVCE reported by Berdugo et al (5), but was weakly positive in one case reported by Xie et al. (11). It was also negative in this case. TLE1 was diffusely and moderately positive in this case, but SS18 was negative. Therefore, the diagnosis of synovial sarcoma could be excluded.

The tissue origin of SCEV has always been a matter of great interest, and some authors have suggested that it originates from the myoepithelium, but this is not supported by the immunohistochemical and ultrastructural features of SCEV (14), nor by the always negative expression of S100. The close mixture of squamous epithelioma, adenoepithelioma, and spindle mesenchymal cells was seen in tumors of Müllerian origin, and the tumor consistently expresses CD10 and BCL2, a feature that resembles the epithelium of Müllerian origin, a viewpoint supported by the fact that this tumor rarely occurs in children and postmenopausal women (3). However, the complex and variable immunophenotype of SCEV is difficult to explain by a single tissue origin; therefore, some scholars have suggested that SCEV originates from multipotent stem cells (6, 7, 14), and the immunohistochemical assays of the present case and other reports show a complex and variable phenomenon, which also supports the hypothesis that SCEV originates from multipotent stem cells.

## Data Availability

The original contributions presented in the study are included in the article/supplementary material. Further inquiries can be directed to the corresponding authors.
